# Serotype distribution, trend of multidrug resistance and prevalence of β-lactamase resistance genes in human *Salmonella* isolates from clinical specimens in Guizhou, China

**DOI:** 10.1371/journal.pone.0282254

**Published:** 2023-04-20

**Authors:** Xiaoyu Wei, Li Long, Lv You, Ming Wang, Dan Wang, Chunting Liu, Shijun Li, Junhua Wang

**Affiliations:** 1 Laboratory of Bacterial Infectious Disease, Guizhou Provincial Center for Disease Control and Prevention, Guiyang, People’s Republic of China; 2 Laboratory of Infectious Disease, Tongren City Center for Disease Control and Prevention, Guiyang, People’s Republic of China; 3 Institute of Communicable Disease Control and Prevention, Guizhou Provincial Center for Disease Control and Prevention, Guiyang, People’s Republic of China; 4 School of Public Health, the Key Laboratory of Environmental Pollution Monitoring and Disease Control, Ministry of Education, Guizhou Medical University, Guiyang, People’s Republic of China; North Carolina State University, UNITED STATES

## Abstract

*Salmonella*, one of the major causes of foodborne infections, can cause bacterial foodborne illness. We investigated the serotype distribution, multidrug resistance (MDR), and β-lactamase resistance genes of human *Salmonella* isolates collected from clinical specimens in Guizhou, China, between 2013 and 2018. A total of 363 *Salmonella* isolates were collected from clinical specimens at 17 surveillance hospitals. Twenty-four serotypes were identified by sliding agglutination test. *S*. Enteritidis (33.9%), *Salmonella* 4,[5],12:i:- (24.0%), *S*. Typhimurium (16.3%), *S*. London (6.3%), and *S*. Derby (3.9%) were the top five serotypes. In 2018, the most common serotype changed from *S*. Enteritidis to *S*. Typhimurium. Among the 363 *Salmonella* isolates, 97.5% of isolates were resistant to at least one class of antimicrobial agents. For cephalosporins, ceftriaxone had the highest resistance rate of 10.5%, and cefepime and cefoxitin were 8.0% and 2.2%, respectively. Three hundred and one (82.9%) *Salmonella* isolates showed MDR. *S*almonella 4,[5],12:i:- had the highest MDR rate with 94.2%, followed by *S*. London (91.3%) and *S*. Typhimurium (88.1%). Multidrug resistance rates of *Salmonella* isolates in Guizhou from 2013 to 2017 increased from 75.8% to 86.7%. Sixteen isolates (4.4%) showed extensive drug resistance. One hundred thirty-four antimicrobial resistance patterns were found. Two hundred and forty-one (66.4%) isolates carried at least one β-lactamase resistance gene. The *bla*_*TEM*_ gene (61.2%) was the most prevalent resistant gene in all *Salmonella* isolates, followed by the *bla*_*CTX-M*_ gene (6.1%) and *bla*_*OXA-1*_ gene (4.1%). Our findings showed that the MDR rate of *Salmonella* isolates from Guizhou province increased year by year. Therefore, systematic and long-term surveillance on MDR *Salmonella* isolates from clinical patients should be further strengthened.

## Introduction

*Salmonella* is one of the most common pathogens of infectious diarrhea worldwide [1]. It is an essential foodborne and zoonotic pathogen. The data from the United States on nontyphoidal *Salmonella* infections indicated that there were approximately 1.2 million illnesses, 23,000 hospitalizations, and 450 deaths each year [2]. In the European Union, the incidence of *Salmonella* infections ranked well behind *Campylobacter* in 2014 [3]. In China, 9.87 million cases of gastroenteritis caused by *Salmonella* each year [4], and outbreak events due to *Salmonella* infection sometimes occurred with the most significant number of patients [5]. The data on bacterial infectious diarrhea disease from the National Infectious Disease Report Management System of China ranked *Salmonella* first in incidence, resulting in 422,325 cases in 2005–2019 [6]. *Salmonella* enterica serovar Enteritidis (*S*. Enteritidis) and *S*. Typhimurium were the first and second serotypes, with 4,140 (36.36%) and 1,502 (13.19%) reported cases, respectively [6]. At the same time, the antimicrobial resistance of *Salmonella* is also one of the most critical public health problems worldwide. Multidrug-resistant (MDR) *Salmonella* poses a severe threat to humans. The MDR of *Salmonella* isolates in the United States was 10.3% from 2004 to 2016 [2]. *Salmonella* isolates from the ten EU member states were tested for nine classes of antimicrobial agents with an overall high MDR(26%) [3]. The level of antibiotic resistance in *Salmonella* varies from country to country and is influenced by antibiotic use practices in humans and animals. *Salmonella* serovars and antimicrobial resistance can display distinct geographic characteristics [7]. From 2011 to 2016, 486 outbreaks of foodborne diseases were reported by various cities and prefectures in Guizhou province through the Foodborne Disease Outbreak Monitoring System. Among the events with clear etiology, *Salmonella* was the primary pathogen causing bacterial foodborne outbreaks and the largest number of cases in Guizhou province [8]. The surveillance results of foodborne diseases in Guizhou province from 2015 to 2017 showed that *Salmonella* ranked first among all pathogens, suggesting that *Salmonella* should be the focus of foodborne disease surveillance in Guizhou province in the future [9]. *S*. Enteritidis was the dominant serotype in infectious diarrheal cases in Guiyang city of Guizhou Province [10]. Furthermore, previous food surveillance in Guizhou province showed that *S*. Typhimurium was the most common serotype [11]. To fully interpret the antimicrobial resistance data, it is necessary to describe the underlying antimicrobial-resistant mechanisms of bacteria. However, previous studies did not provide sufficient information on the systematic serotype distribution, the trend of MDR, and the prevalence of β-lactamase resistance gene in clinical *Salmonella* isolates in Guizhou province. Few data on the trends of historical antimicrobial resistance in *Salmonella* isolates are available.

This study investigated the serotype distribution, the trend of MDR, and the prevalence of β-lactamase resistance genes of *Salmonella* isolates from clinical specimens in Guizhou, with a special focus on the consistency between the presence of the β-lactamase resistance gene and β-lactamase antimicrobial phenotypes. These results could provide a reference basis for the scientific prevention and control of *Salmonella* infection and the rational use of antimicrobials.

## Materials and methods

### Ethics statement

This study was a retrospective study of archived samples. We ensured that all data were fully anonymized. The present study was reviewed and approved by the Ethics Review Committee of Guizhou Provincial Center for Disease Control and Prevention.

### Isolates collection and identification

All the cities, including six cities and three autonomous prefectures in Guizhou province of China, were included in this program. Patients with the following symptoms were selected for the study: Three or more episode of diarrhea within 24 hours, watery or sticky stools, mucus or pus-bloody stools. Also, the following criteria were considered for suspicious cases of non-typhoidal *Salmonella* infection: fever (temperature >38°C), or with headache, chills, fatigue; nausea, vomiting or abdominal pain. Stool samples from patients with clinical diarrhea were collected to isolate *Salmonella* from 2013 to 2018. All fecal samples were cultured overnight at 37°C in local hospitals using MacConkey agar plates (Huankai, Guangdong, China). Systematic biochemical methods were used to identify suspected colonies [12]. All suspicious *Salmonella* isolates were submitted to the laboratory of Guizhou Provincial Center for Disease Control and Prevention (Guizhou CDC) for further validation and serotyping. In the laboratory, these isolates were recovered by inoculating on nutrient agar plates (Huan Kai, Guangdong, China) and further identified by the API20E identification kit (Biomerieux, France). Identified *Salmonella* isolates were serotyped by O and H antigen slide agglutination tests (SSI, Denmark) according to the White-Kauffmann-Le Minor scheme [13].

### Antimicrobial resistance test

The antimicrobial resistance of all *Salmonella* isolates was examined using previously reported the method of micro broth dilution (Xingbai, Shanghai) [1]. Sixteen antimicrobial agents of ten classes were tested, including Penicillin (ampicillin), Phenicols (chloramphenicol), Aminoglycosides (streptomycin, gentamicin), Carbapenems (imipenem), β-lactamase inhibitor (amoxicillin/clavulanic acid), Cephems (cefoxitin, ceftriaxone, cefepime), Sulfonamides (sulfamethoxazole, trimethoprim-sulfamethoxazole), Tetracyclines (tetracycline, doxycycline), Quinolones and Fluoroquinolones (nalidixic acid, ciprofloxacin), Macrolides (azithromycin). Different antimicrobial breakpoints were interpreted by Clinical Laboratory Standards Institute guidelines [14]. Streptomycin (STR) was interpreted by the National Antimicrobial Resistance Monitoring System for enteric bacteria (NARMS) established breakpoints for *Salmonella* isolates (https://www.cdc.gov/narms/antibiotics-tested.html). Escherichia coli ATCC 25922 was used as a control strain. *Salmonella* isolates resistant to at least three different classes of antimicrobials were defined as MDR, while *Salmonella* isolates resistant to at least eight different classes of antimicrobials were defined as extensively drug resistant (XDR).

### Detection of β-lactamase resistance genes

All *Salmonella* isolates were screened for seven *β*-lactamase genes (*bla*_TEM_, *bla*_SHV_, *bla*_OXA-1_, *bla*_OXA-2_, *bla*_PSE_, *bla*_CMY_, and *bla*_CTX-M_) using a simplex PCR assay. The primers used to amplify the antimicrobial resistance genes (ARGs) in this study were listed in S1 Table. The PCR assay was performed according to the reported reaction conditions [15, 16]. The isolates positive for *bla*_TEM_ and *bla*_CTX-M_ genes were further sent for sequencing. The DNA sequences obtained were further aligned using BLAST analysis to identify the subtypes of resistance genes (https://blast.ncbi.nlm.nih.gov/Blast.cgi).

### Statistical analysis

Statistical analysis was performed using SPSS statistical software package (version 22.0; IBM, Chicago, USA). The chi-square test was used to compare the prevalence of *Salmonella* isolates and antimicrobial resistance rates in different cities. The consistency and Kappa were used to analyze the concordance between the presence of the β-lactamase an and β-lactamase antimicrobial phenotypes. A *P* value of <0.05 was define as statistical significance.

## Results

### Serotypes distribution of *Salmonella* isolates

**Fig 1:**
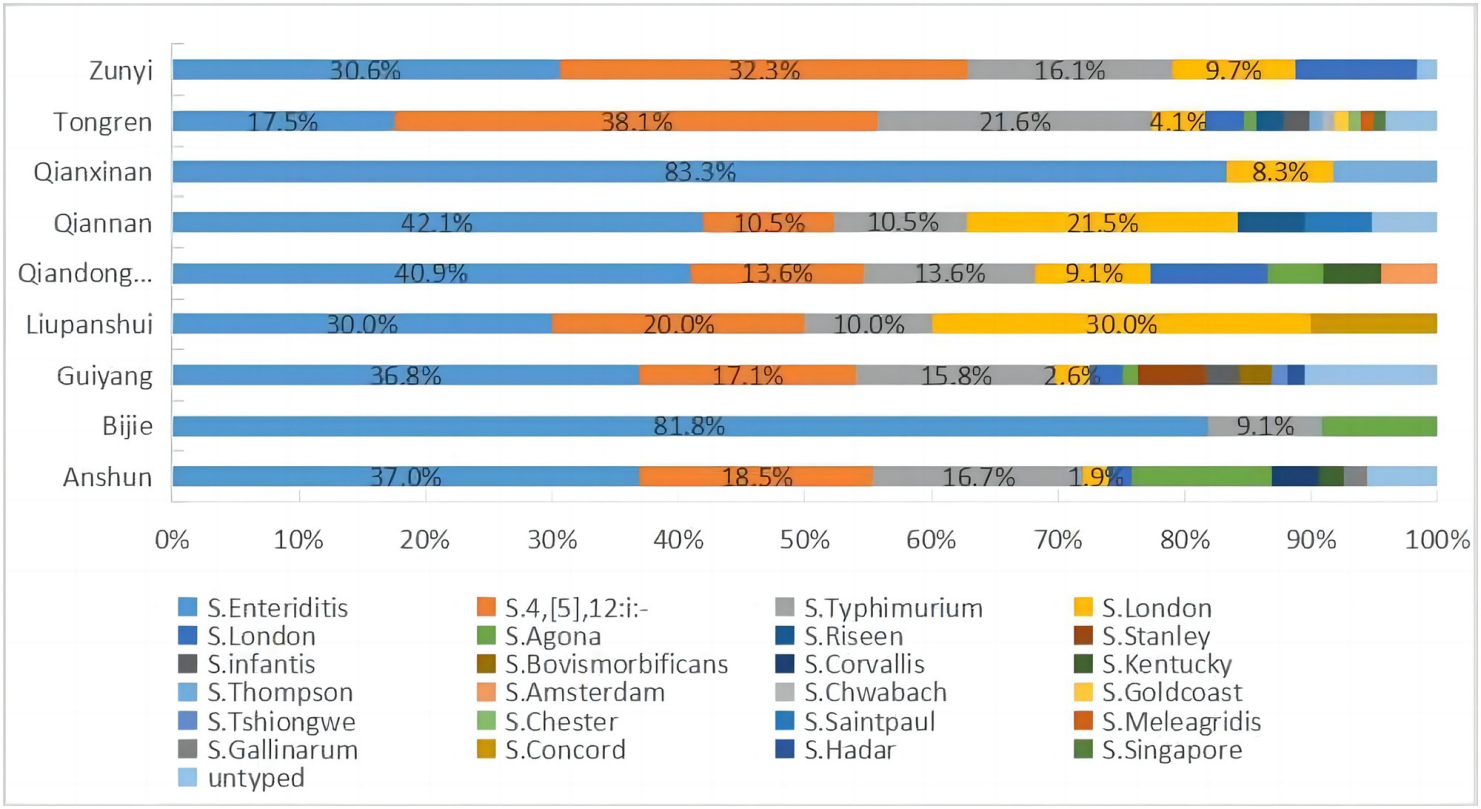
The serotype distribution of *Salmonella* isolates from six cities and three autonomous prefectures in Guizhou province between 2013 and 2018.

A total of 363 *Salmonella* isolates from clinical specimens were submitted to the laboratory of Guizhou CDC during 2013–2018. The proportion of *Salmonella* isolates was highest in Tongren city (26.7%), followed by Guiyang city (20.9%), Zunyi city (17.1%), Anshun city (14.9%), Qiandongnan prefecture (6.1%), Qiannan prefecture (5.2%), Qianxinan prefecture (3.3%), Bijie city (3.0%), and Liupanshui city (2.8%). Twenty-four serotypes were identified among 346 *Salmonella* isolates. However, 17 *Salmonella* isolates could not be determined by serotype (Table 1). *S*. Enteritidis (123, 33.9%) was the most common serotype, followed by *Salmonella* 4,[5],12:i:- (87, 24.0%), *S*. Typhimurium (59, 16.3%), *S*. London (23, 6.3%), and *S*. Derby (14, 3.9%). These five serotypes were composed of 316 (87.1%) isolates. The distribution of dominant serotypes had changed between 2013 and 2018. *S*. Enteritidis and *Salmonella* 4,[5],12: i:- ranked first and second, respectively, from 2013 to 2017, while *S*. Typhimurium surpassed the above two serotypes to become the dominant serotype in 2018. In addition, the number of *Salmonella* serotypes increased from 10 in 2013 to 15 in 2018. The geographical distribution of *Salmonella* serotypes among the clinical samples was shown in Fig 1. *Salmonella* 4,[5],12:i:- was the dominant serotype in Zunyi and Tongren. However, *S*. Enteritidis was the predominant serotype in the other seven cities (prefectures), including Qiannan, Qianxinan, Bijie, Anshun, Guiyang, Liupanshui, and Qiandongnan. Notably, *Salmonella* isolates from Tongren and Guiyang were more diverse, with 15 and 12 serotypes, respectively (Fig 1).

**Table 1:** The serotype distribution of human *Salmonella* isolates in Guizhou province, China, from 2013 to 2018.

Serotype	Number of isolates by year (recovery rate, %)						
	2013 (*n* = 33)	2014(*n* = 33)	2015(*n* = 34)	2016(*n* = 72)	2017(*n* = 105)	2018(*n* = 85)	Total(*n* = 363)
*S*. Enteritidis	11(33.3)	15(45.5)	12(35.3)	29(40.3)	36(34.3)	20(23.3)	123(33.9)
*S*. 4, [5], 12: i: -	7(21.2)	6(18.2)	10(29.4)	18(25.0)	27(25.7)	19(22.1)	87(24)
*S*. Typhimurium	3(9.1)	3(9.1)	1(2.9)	11(15.3)	17(16.2)	24(27.9)	59(16.3)
*S*. London	0(0.0)	1(3.0)	0(0.0)	7(9.7)	10(9.5)	5(5.8)	23(6.3)
*S*. Derby	3(9.1)	0(0.0)	1(2.9)	1(1.4)	6(5.7)	3(3.5)	14(3.9)
*S*. Agona	1(3.0)	0(0.0)	5(14.7)	0(0.0)	0(0.0)	3(3.5)	9(2.5)
*S*. Rosen	0(0.0)	0(0.0)	0(0.0)	1(1.4)	2(1.9)	1(1.2)	4(1.1)
*S*. Stanley	2(6.1)	1(3.0)	0(0.0)	1(1.4)	0(0.0)	0(0.0)	4(1.1)
*S*. Infantis	1(3.0)	1(3.0)	1(2.9)	0(0.0)	1(1.0)	0(0.0)	4(1.1)
*S*. bovis morbificans	1(3.0)	0(0.0)	0(0.0)	0(0.0)	0(0.0)	1(1.2)	2(0.6)
*S*. Corvallis	0(0.0)	0(0.0)	1(2.9)	0(0.0)	0(0.0)	1(1.2)	2(0.6)
*S*. Kentucky	0(0.0)	0(0.0)	0(0.0)	0(0.0)	2(1.9)	0(0.0)	2(0.6)
*S*. Thompson	0(0.0)	0(0.0)	0(0.0)	2(2.8)	0(0.0)	0(0.0)	2(0.6)
*S*. Amsterdam ii	0(0.0)	0(0.0)	0(0.0)	0(0.0)	1(1.0)	0(0.0)	1(0.3)
*S*. Chwabach	0(0.0)	0(0.0)	0(0.0)	0(0.0)	0(0.0)	1(1.2)	1(0.3)
*S*. Gold Coast	0(0.0)	0(0.0)	0(0.0)	0(0.0)	0(0.0)	1(1.2)	1(0.3)
*S*. Tsangwi	0(0.0)	0(0.0)	0(0.0)	0(0.0)	1(1.0)	0(0.0)	1(0.3)
*S*. Chester	0(0.0)	0(0.0)	1(2.9)	0(0.0)	0(0.0)	0(0.0)	1(0.3)
*S*. Saintpaul	0(0.0)	0(0.0)	0(0.0)	0(0.0)	0(0.0)	1(1.2)	1(0.3)
*S*. Turkey	0(0.0)	0(0.0)	0(0.0)	0(0.0)	0(0.0)	1(1.2)	1(0.3)
*S*. Gallinarum	0(0.0)	0(0.0)	1(2.9)	0(0.0)	0(0.0)	0(0.0)	1(0.3)
*S*. Concord	0(0.0)	0(0.0)	0(0.0)	0(0.0)	1(1.0)	0(0.0)	1(0.3)
*S*. Hadar	0(0.0)	0(0.0)	0(0.0)	0(0.0)	0(0.0)	1(1.2)	1(0.3)
*S*. Singapore	0(0.0)	0(0.0)	0(0.0)	0(0.0)	0(0.0)	1(1.2)	1(0.3)
Unclassified	4(12.1)	6(18.2)	1(2.9)	2(2.8)	1(1.0)	3(3.5)	17(4.7)

### Antimicrobial resistance changes

Antimicrobial resistance testing of the 363 *Salmonella* isolates revealed that 354 (97.5%) isolates were resistant to at least one class of antimicrobial agent. The highest resistance rate was observed for sulfamethoxazole (86.0%), followed by streptomycin (81.5%) and ampicillin (76.0%) (Table 2). For ceftriaxone, cefepime, and cefoxitin, the resistance rates were 10.5%, 8.0%, and 2.2%, respectively. Furthermore, some *Salmonella* isolates showed intermediate resistance, such as ciprofloxacin (65.0%), chloramphenicol (17.9%), and amoxicillin/clavulanic acid (17.9%). The antimicrobial resistance of the top five serotypes, including *S*. Enteritidis, *S*. Typhimurium, *Salmonella* 4,[5],12:i:-, *S*. London, and *S*. Derby, was shown to be highly resistant to streptomycin (77.8%-95.7%) and sulfamethoxazole (71.4%-91.3%). The antimicrobial resistance rate of *S*. Enteritidis to chloramphenicol, trimethoprim-sulfamethoxazole, doxycycline, and tetracycline was lower than that of the other four serotypes (P < 0.005) (Table 2), while its antimicrobial resistance rate to nalidixic acid was the highest. The antimicrobial resistance rate of *S*. London to gentamicin and azithromycin was higher than that of the other four serotypes (P < 0.005), and the drug resistance rate to ciprofloxacin was higher than that of *S*. Enteritidis and *S*. Typhimurium (P < 0.005) (Table 2). Although the resistance rate of *S*. Enteritidis to ciprofloxacin was only 1.6%, the intermediate resistance to ciprofloxacin in *S*. Enteritidis was extremely high at 96.0%. Meanwhile, *S*. Enteritidis exhibited higher resistance to nalidixic acid (97.6%) than the other four serotypes. *Salmonella* 4,[5],12:i:- showed higher resistance to the third-generation cephalosporin (ceftriaxone) than the other four serotypes.

**Table 2:** Antimicrobial resistance of the top five *Salmonella* serotypes isolates from humans in Guizhou province, China, from 2013 to 2018.

Antimicrobials	R (%)	I (%)	S (%)	Number of isolates (%)					χ^2^	*P*
				*S*. Enteritidis (*n* = 123)	*S*. 4,[5],12:i:-(n = 87)	*S*. Typhimurium (*n* = 59)	*S*. London (*n* = 23)	*S*. Derby (*n* = 14)		
CIP	42(11.6)	236(65.0)	85(23.4)	2(1.6)	12(13.8)	6(10.2)	9(39.1)	2(14.3)	35.564	<0.001
STR	296(81.5)	0(0.0)	67(18.5)	98(79.8)	79(90.8)	50(84.7)	22(95.7)	11(78.6)	7.525	0.111
AM	276(76.0)	0(0.0)	87(24.0)	98(79.8)	77(88.5)	48(81.4)	21(91.3)	9(64.3)	7.412	0.116
C	122(33.6)	65(17.9)	176(48.5)	6(4.8)	37(42.5)	44(74.6)	12(52.2)	9(64.3)	101.522	<0.001
SOX	312(86.0)	0(0.0)	51(14.0)	104(84.7)	81(93.1)	52(88.1)	21(91.3)	10(71.4)	7.145	0.128
SXT	137(37.7)	0(0.0)	226(62.3)	17(13.8)	31(35.6)	41(69.5)	21(91.3)	8(57.1)	84.963	<0.001
NAL	192(52.9)	0(0.0)	171(47.1)	120(97.6)	30(34.5)	15(25.4)	4(17.4)	5(35.7)	141.758	<0.001
AMC	84(23.1)	65(17.9)	214(59.0)	32(26.0)	27(31.0)	12(20.3)	0(0.0)	1(7.1)	12.646	0.013
CRO	38(10.5)	3(0.8)	322(88.7)	8(6.5)	17(19.5)	4(6.8)	0(0.0)	1(7.1)	14.064	0.007
DOX	210(57.9)	19(5.2)	134(36.9)	28(22.8)	75(86.2)	48(81.4)	18(78.3)	13(92.9)	116.132	<0.001
GEN	53(14.6)	5(1.4)	305(84.0)	5(4.1)	16(18.4)	7(11.9)	19(82.6)	1(7.1)	107.714	<0.001
AZM	33(9.1)	0(0.0)	330(90.9)	4(3.3)	5(5.7)	2(3.4)	16(69.6)	0(0.0)	114.771	<0.001
TE	220(60.6)	10(2.8)	133(36.6)	35(28.5)	75(86.2)	47(79.7)	19(82.6)	14(100.0)	101.016	<0.001
FOX	8(2.2)	4(1.1)	351(96.7)	0(0.0)	1(1.1)	1(1.7)	0(0.0)	0(0.0)	3.804	0.454
FEP	29(8.0)	7(1.9)	327(90.1)	7(5.7)	13(14.9)	3(5.1)	1(4.3)	1(7.1)	7.542	0.11
IPM	6(1.7)	0(0.0)	357(98.3)	2(1.6)	1(1.1)	2(3.4)	1(4.3)	0(0.0)	2.536	0.614

Abbreviation: ciprofloxacin (CIP), Streptomycin (STR), ampicillin (AM), chloramphenicol (C), sulfamethoxazole (SOX), trimethoprim-sulfamethoxazole (SXT), nalidixic acid (NAL), amoxicillin/clavulanate (AMC), ceftriaxone (CRO), doxycycline (DOX), gentamicin (GEN), azithromycin (AZM), tetracycline (TE), cefoxitin (FOX), cefepime (FEP), and imipenem (IPM)

Antimicrobial resistance changes of human *Salmonella* isolates in Guizhou province from 2013 to 2018 were analyzed (Table 3). The results showed that the antimicrobial resistance rate of *Salmonella* isolates to ampicillin, chloramphenicol, trimethoprim-sulfamethoxazole, doxycycline, tetracycline, cefoxitin, and imipenem was on the increase (P < 0.05). While the trends for resistance rates to sulfamethoxazole, nalidixic acid and amoxicillin/ clavulanic acid were on the decrease (P < 0.05). The antimicrobial resistance rates of the isolates to the third/fourth-generation cephalosporins ranged from 5.8% to 19.4%.

**Table 3:** Antimicrobial resistance of *Salmonella* isolates in Guizhou from 2013 to 2018.

Antimicrobials	Number of isolates (%)						χ^2^	*P*
	2013 (*n* = 33)	2014 (*n* = 33)	2015 (*n* = 34)	2016 (*n* = 72)	2017 (*n* = 105)	2018 (*n* = 86)		
CIP	3(9.1)	2(6.1)	1(2.9)	11(15.3)	9(8.6)	16(18.6)	3.536	0.06
STR	28(84.8)	27(81.8)	32(94.1)	53(73.6)	85(81.0)	71(82.6)	0.303	0.582
AM	19(57.6)	23(69.7)	23(67.6)	57(79.2)	88(83.8)	66(76.7)	7.383	0.007
C	6(18.2)	5(15.2)	6(17.6)	17(23.6)	41(39.0)	47(54.7)	28.012	<0.001
SOX	32(97.0)	29(87.9)	32(94.1)	61(84.7)	90(85.7)	68(79.1)	6.562	0.01
SXT	10(30.3)	10(30.3)	3(8.8)	23(31.9)	47(44.8)	44(51.2)	13.512	<0.001
NAL	21(63.6)	19(57.6)	18(52.9)	42(58.3)	56(53.3)	36(41.9)	4.617	0.032
AMC	17(51.5)	24(72.7)	23(67.6)	10(13.9)	3(2.9)	7(8.1)	94.933	<0.001
CRO	2(6.1)	3(9.1)	4(11.8)	14(19.4)	10(9.5)	5(5.8)	0.204	0.651
DOX	15(45.5)	17(51.5)	19(55.9)	38(52.8)	62(59.0)	59(68.6)	6.248	0.012
GEN	3(9.1)	2(6.1)	1(2.9)	17(23.6)	14(13.3)	16(18.6)	3.815	0.051
AZM	1(3.0)	1(3.0)	0(0.0)	14(19.4)	9(8.6)	8(9.3)	2.258	0.133
TE	16(48.5)	16(48.5)	19(55.9)	39(54.2)	65(61.9)	65(75.6)	11.12	0.001
FOX	0(0.0)	2(6.1)	0(0.0)	3(4.2)	2(1.9)	1(1.2)	129.474	<0.001
FEP	2(6.1)	2(6.1)	4(11.8)	9(12.5)	7(6.7)	5(5.8)	0.157	0.692
IPM	0(0.0)	0(0.0)	1(2.9)	3(4.2)	0(0.0)	2(2.3)	12.64	<0.001

Abbreviation: ciprofloxacin (CIP), Streptomycin (STR), ampicillin (AM), chloramphenicol (C), sulfamethoxazole (SOX), trimethoprim-sulfamethoxazole (SXT), nalidixic acid (NAL), amoxicillin/clavulanate (AMC), ceftriaxone (CRO), doxycycline (DOX), gentamicin (GEN), azithromycin (AZM), tetracycline (TE), cefoxitin (FOX), cefepime (FEP), and imipenem (IPM)

### Trend of multidrug resistance

**Fig 2:**
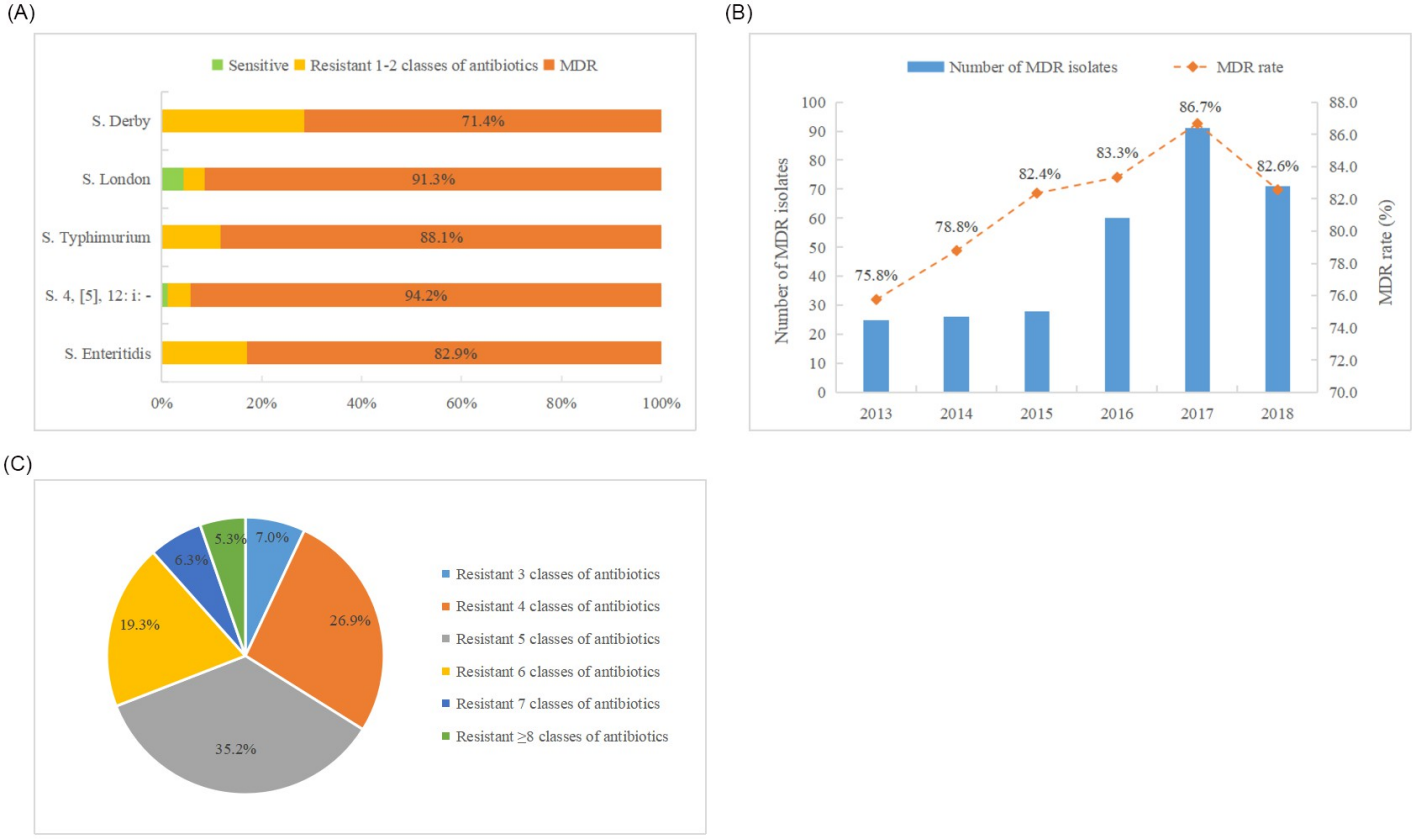
(A) MDR of the top five serotypes of *Salmonella* isolates in Guizhou, (B) MDR rates of *Salmonella* isolates in Guizhou from 2013 to 2018, (C) Resistant to ≥ three classes of antibiotics for *Salmonella* isolates in Guizhou from 2013 to 2018.

Among the 363 *Salmonella* isolates, 301 (82.9%) *Salmonella* isolates showed MDR. The proportion of MDR isolates in the top five serotypes was high. *Salmonella* 4,[5],12:i:- and *S*. London had the highest MDR rate with 94.2% (82/87) and 91.3% (21/23), respectively (Fig 2A). At the same time, the rate of MDR increased from 75.8% to 86.7% during the years 2013 to 2017, which showed a trend of increasing year by year, except for 2018 (Fig 2B). The MDR of clinical *Salmonella* isolates from nine cities (prefectures) in Guizhou between 2013 and 2018 showed that the highest rate of MDR was observed in Tongren city with 90.7% (88/93). The higher rates of MDR were found in other cities (prefectures) with 72.7%-90.0%. Among the MDR *Salmonella* isolates, the resistance to five classes of antibiotics (35.2%, 106/301) was dominant, followed by resistance to four classes (26.9%, 81/301), and six classes (19.3%, 58/301) (Fig 2C). In addition, 16 isolates (5.3%, 16/301) were resistant to more than eight classes of antibiotics and showed extensive drug resistance (S2 Table).

One hundred and thirty-four AMR patterns among 363 *Salmonella* isolates to ten classes of antimicrobials were identified (S2 Table). The dominant AMR pattern was AM+STR+SOX+NAL (11.3%, 41/363), followed by AM+C+STR+SOX+TE+DOX+SXT (8.0%, 29/363) and AM+STR+SOX+NAL+AMC (4.7%, 17/363). This study analyzed the AMR patterns of the top five serotypes (S3 Table). Thirty-nine distinctive AMR patterns of *S*. Enteritidis were found, and the dominant AMR pattern was AM+STR+SOX+NAL (32.5%). Forty-six different AMR patterns of 87 *Salmonella* 4,[5],12:i:- isolates were observed, and the dominant AMR patterns were AM+STR+SOX+TE+DOX+AMC (16.1%) and AM+STR+SOX+TE+DOX (14.9%). Thirty distinctive AMR patterns of *S*. Typhimurium were found, and the dominant AMR pattern was AM+C+STR+SOX+TE+DOX+SXT (32.2%). Among these common serotypes, *S*. Typhimurium, *S*. London, *S*. Derby, and *Salmonella* 4,[5],12:i:- were resistant to ACSSuT (AM+C+STR+SOX+TE) with a proportion of 57.6%, 52.2%, 64.3%, and 35.6% respectively, and only six *S*. Enteritidis isolates were resistant to ACSSuT.

### Detection of β-lactamase antimicrobial resistance genes

PCR results indicated that 241 *Salmonella* isolates (66.4%) carried at least one β-lactamase resistance gene. The most prevalent was the *bla*_*TEM*_ gene (61.2%, 222/363), followed by the *bla*_*CTX-M*_ gene (6.1%, 22/363) and *bla*_OXA-1_ gene (4.1%, 15/363) (Table 4). Ten (2.8%) isolates produced both *bla*_*TEM*_ and *bla*_*CTX-M*_ genes, and one isolates carried *bla*_*TEM*_, *bla*_*CTX-M*_, and *bla*_*OXA-1*_ genes simultaneously. The *bla*_*SHV*_, *bla*_*OXA-2*_, and *bla*_*PER*_ genes were not detected in the *Salmonella* isolates. According to sequence BLAST online, the 222 *Salmonella* isolates with the *bla*_*TEM*_ gene belonged to *bla*_*TEM-1*_. Seven subtypes of the *bla*_*CTX-M*_ gene were obtained in the 22 *bla*_*CTX-M*_ gene-positive isolates, of which the prevalent subtypes were *bla*_*CTX-M-55*_ (68.2%, 15/22) and *bla*_*CTX-M-65*_ (9.1%, 2/22). *Bla*_*CMY*_ gene was detected in two *Salmonella* isolates and identified as *bla*_*CMY-2*_. The two isolates carrying the *bla*_*CMY-2*_ gene were resistant to more than ten classes of antibiotics, including β-lactamase antibiotics, such as ceftriaxone, cefepime, ampicillin, amoxicillin/clavulanate.

**Table 4:** The prevalence of β-lactamase resistance genes of *Salmonella* isolates.

β-lactamase resistance genes	Subtypes of genes	Number of isolates	Percent (%)
*bla*_*TEM*_	*bla*_*TEM-1*_	222	61.2
*bla*_*CTX-M*_	*bla*_*CTX-M-55*_	15	4.1
	*bla*_*CTX-M-65*_	2	0.6
	*bla*_*CTX-M-14*_	1	0.3
	*bla*_*CTX-M-15*_	1	0.3
	*bla*_*CTX-M-27*_	1	0.3
	*bla*_*CTX-M-64*_	1	0.3
	*bla*_*CTX-M-153*_	1	0.3
*bla*_*CMY*_	*bla*_*CMY-2*_	2	0.6

*Salmonella* isolates with different serotypes differed in carrying the β-lactamase resistance gene. *Bla*_*TEM*_ gene was observed in 14 serotypes of *Salmonella* isolates. The top three serotypes were *S*. Enteritidis (60.9%), *Salmonella* 4,[5],12:i:- (73.6%), and *S*. Typhimurium (69.5%). *Bla*_*CTX-M*_ gene was identified in *S*. Enteritidis (1.6%), *Salmonella* 4,[5],12:i:- (13.8%), and *S*. Typhimurium (6.8%). Among them, *S*. Enteritidis only carried *bla*_*CTX-M-55*_. Five subtypes of the *bla*_*CTX-M*_ gene were obtained in 12 *Salmonella* 4,[5],12:i:- isolates, of which the prevalent gene was *bla*_*CTX-M-55*_. Meanwhile, three subtypes of the *bla*_*CTX-M*_ gene were obtained in four *S*. Typhimurium isolates.

The prevalence of β-lactamase resistance genes of *Salmonella* isolates varied among regions. The detection rate of *bla*_*TEM*_ gene in each city (prefecture) ranged from 44.4% to 81.5%, with the highest detection rate in Bijie. *Bla*_*OXA-1*_ gene was detected in five cities (prefectures), including Bijie, Liupanshui, Guiyang, Tongren and Zunyi, with the highest detection rate in Tongren (9.3%). *Bla*_*CTX-M*_-positive strains were distributed in six cities (prefectures), including Anshun, Guiyang, Qiandongnan, Qianxinan, Tongren and Zunyi, with the highest detection rate in Bijie (9.1%). The *bla*_*CMY*_ gene was only detected in *Salmonella* strains in Tongren.

### The concordance between the β-lactamase ARGs and phenotypic antimicrobial resistant

In this study, we compared phenotypic and genotypic resistance to five β-lactamase antimicrobials. There was an overall concordance of 80.4% between phenotypic and genotypic resistance against 363 *Salmonella* isolates. Of these 279 phenotype-positive *Salmonella* isolates, 227 (81.3%) contained the β-lactamase resistance genes. However, of 84 phenotype-negative *Salmonella* isolates, 19 (22.6%) contained the β-lactamase resistance genes. A certain correlation was found between the *bla*_*TEM*_ gene and ampicillin (Kappa = 0.452, P < 0.001). Furthermore, the *bla*_*CTX-M*_ gene was in good agreement with ceftriaxone and cefepime (Kappa = 0.688, Kappa = 0.655, P < 0.001), as shown in Table 5.

**Table 5:** The concordance between β-lactamase ARGs and phenotypic antimicrobial resistance of *Salmonella* isolates.

ARGs VS antimicrobials	ARGs-positive		ARGs-negative		Kappa	*P*
	phenotypic antimicrobial resistant-positive	phenotypic antimicrobial resistant-negative	phenotypic antimicrobial resistant-positive	phenotypic antimicrobial resistant-negative		
*bla*_*TEM*_ vs AM	207	18	69	69	0.452	<0.001
*bla*_*TEM*_ vs AMC	56	169	31	107	0.02	0.599
*bla*_*TEM*_ vs CRO	18	207	20	118	-0.051	0.05
*bla*_*TEM*_ vs FEP	15	210	16	122	-0.039	0.103
*bla*_*TEM*_ vs FOX	2	223	5	133	-0.021	0.066
*bla*_*OXA-1*_ vs AM	14	1	262	86	0.019	0.109
*bla*_*OXA-1*_ vs AMC	7	8	80	268	0.072	0.057
*bla*_*OXA-1*_ vs CRO	4	11	34	314	0.097	0.06
*bla*_*OXA-1*_ vs FEP	2	13	29	319	0.033	0.372
*bla*_*OXA-1*_ vs FOX	3	12	4	334	0.253	0.002
*bla*_*CTX-M*_ vs AM	21	1	255	86	0.032	0.028
*bla*_*CTX-M*_ vs AMC	8	14	79	262	0.055	0.16
*bla*_*CTX-M*_ vs CRO	21	1	16	324	0.688	<0.001
*bla*_*CTX-M*_ vs FEP	18	4	13	328	0.655	<0.001
*bla*_*CTX-M*_ vs FOX	0	22	7	334	-0.03	1
*bla*_*CMY*_ vs AMP	2	0	274	87	0.003	1
*bla*_*CMY*_ vs AMC	2	0	85	276	0.035	0.057
*bla*_*CMY*_ vs CRO	2	0	36	325	0.09	0.11
*bla*_*CMY*_ vs FEP	0	2	31	330	-0.01	1
*bla*_*CMY*_ vs FOX	2	0	5	356	0.34	<0.001

*Abbreviation*: ampicillin (AM), amoxicillin/clavulanate (AMC), ceftriaxone (CRO), cefepime (FEP), cefoxitin (FOX).

## Discussion

Serological identification in this study showed that the number of *Salmonella* isolates in the top ten serotypes accounted for more than 90%, among which *S*. Enteritidis, *Salmonella* 4,[5],12: i:- and *S*. Typhimurium were the main serotypes. The prevalence of serotypes was similar to those reported in the United States [17], the European Union [18], and Guangdong Province [19]. However, the serotypes were different from the dominant serotype of *S*. Agona reported in Maanshan city, Anhui province [20]. This information indicated that the distribution of *Salmonella* serotypes had regional characteristics. *S*. Enteritidis is mainly associated with contaminated eggs, while *Salmonella* 4,[5],12:i:- and *S*. Typhimurium are mainly associated with contaminated pork and pork products. These contaminated foods are easily transmitted to humans through the food chain and cause illness. Future surveillance in Guizhou province should be conducted to determine the source of *Salmonella* infections and risk factors for disease control and prevention. In this study, *Salmonella* serotypes became more and more diverse from 2013 to 2018. Meanwhile, 17 isolates could not be classified by serotype, and either new serotypes or other rare serotypes require further investigation. The continued emergence of rare serotypes of *Salmonella* should be of great concern, as these *Salmonella* serotypes have the potential to spread and cause public health problems. These results indicate that the dynamic monitoring of *Salmonella* serotypes and focusing on serotype changes in different regions are significant for targeting the prevention and control of salmonellosis in Guizhou.

Increasing antimicrobial resistance was found in this study, especially to the traditional agents, such as sulfamethoxazole, streptomycin, ampicillin, tetracycline, doxycycline, and nalidixic acid, which was similar to the results reported in some regions of China [21, 22], but greatly higher than those of human *Salmonella* in the European Union [23]. Decreased susceptibility to ciprofloxacin is a current trend in non-typhoidal *Salmonella* isolates worldwide [24]. In this study, decreased susceptibility to ciprofloxacin was as high as 65.0% (MIC≥0.12μg/mL), which may affect clinical treatment and may lead to treatment failure [24]. Therefore, ciprofloxacin sensitivity should be carefully considered when selecting this antimicrobial agent. MDR *Salmonella* has become a significant threat to human health. The overall rate of MDR (82.9%) was much higher than that reported in Zhejiang and Anhui, China [25, 26]. MDR *Salmonella* increased from 2013 to 2017 in this study, possibly as a result of antimicrobial misuse in humans. In addition, MDR was widely distributed in various serotypes of *Salmonella* in this study, especially in *Salmonella* 4,[5],12: i:- (94.2%) and *S*. London (91.3%), which were much higher than the results reported in other cities [27–29]. In addition, 5.3% of the isolates showed XDR and 16 isolates were resistant to ten or more antibiotics simultaneously. In terms of regional distribution, Tongren city had the highest MDR rate, followed by Liupanshui city. Meanwhile, we discovered that the AMR patterns of *Salmonella* isolates became more diverse and broader year by year. Our results highlight the serious issue of *Salmonella* MDR in clinical samples in Guizhou, which may lead to the evolution of *Salmonella* into a super bacterium and pose a risk to public health [30, 31]. Therefore, the enhanced and continuous surveillance of antimicrobial resistance *Salmonella* in different serotypes and regions should be carried out to further monitor MDR *Salmonella* with public health concerns. Appropriate and effective antimicrobial agents for the treatment of *Salmonella* infection should be selected according to drug sensitivity testing in each region.

Moreover, a total of four β-lactamase resistance genes were detected in this study, among which *bla*_*TEM*_ had the highest detection rate (61.2%), which was similar to the results of human *Salmonella* studies in Zhejiang [32] and Beijing [33], but different from *bla*_*OXA-1*_ prevalent in Henan [34] and *bla*_*CTX-M*_ prevalent in Shanghai [35]. This result may be related to the different types of antibiotics used and the prevalence of serotypes in different regions. It is noteworthy that 130 *Salmonella* isolates from nine regional pig farms in Guizhou province were detected for β-lactamase resistance genes, of which *bla*_*TEM*_ with a detection rate of 85% was consistent with the results of this study [36]. Pork and pork production may be the source of β-lactamase resistance genes in Guizhou province. All the *bla*_*TEM*_-positive isolates in this study were harbored *bla*_*TEM-1*_ gene, which was different from *bla*_*TEM-52*_, *bla*_*TEM-3*_, and *bla*_*TEM-27*_ *genes* prevalent in other countries [37–39]. In addition, we noticed that *bla*_*TEM*-1_ isolates were widely distributed among different serotypes, suggesting that this genotype had no serotype specificity and might be continuously spreading in *Salmonella* isolates from Guizhou province. Therefore, we should further strengthen the β-lactamase resistance gene surveillance of *Salmonella* isolates from clinical samples and pork production.

In this study, 6.1% of isolates carried the *bla*_*CTX-M*_ gene, which was lower than the detection rate of *bla*_*CTX-M*_ reported in Zhejiang [32]. *Bla*_*CTX-M*_ gene subtypes were diverse, and the most common subtype was *bla*_*CTX-M-55*_, which was consistent with other reports such as Sichuan [40] and Guangdong [41]. The above two provinces are closer to Guizhou province, and there may be the cross-regional transmission. However, *bla*_*CTX-M-14*_ and *bla*_*CTX-M-15*_ were prevalent in Jiangxi [42] and Beijing [33]. The distribution of *bla*_*CTX-M*_ showed significant regional differences. Meanwhile, we found that *bla*_*CTX-M*_ was mainly distributed in a few serotypes, especially *Salmonella* 4,[5],12: i:-, in which more isolates carried the *bla*_*CTX-M*_ gene with diverse subtypes. This is consistent with the higher resistant rate of *Salmonella* 4,[5],12:i:- to cephalosporins than other serotypes in this study.

Bacterial production of the AmpC enzyme is another major resistance mechanism of Gram-negative bacilli to β-lactam antibiotics following ESBLs in recent years [43]. Newly discovered plasmid-mediated AmpC enzymes have gradually increased, and *Bla*_*CMY*_-producing *Salmonella* has continued to spread in North America, becoming a major concern for cephalosporin resistance in this region [37]. Ceftriaxone-resistant *Salmonella* isolates in the United States were mainly mediated by the plasmid-encoded *bla*_*CMY*_ gene [44]. Plasmid-encoded *bla*_*CMY-2*_ was the most common and destructive β-lactamase, which can seriously affect the therapeutic effect of broad-spectrum cephalosporins [34]. In this study, two isolates with the *bla*_*CMY*_ gene were detected and identified as *bla*_*CMY-2*_ by sequencing comparison, which was reported for the first time in Guizhou. The two *bla*_*CMY-2*_-positive clinical *Salmonella* isolates were resistant to ceftriaxone, cefepime, ciprofloxacin, azithromycin, and other clinically significant antibiotics. We noticed that isolates carrying *bla*_*CMY-2*_ *gene* were usually multidrug resistant, which puts tremendous pressure on the prevention and control of multidrug resistant bacteria [45, 46]. Therefore, it is essential to strengthen the surveillance of *bla*_*CTX-M*_ and *bla*_*CMY-2*_ positive *Salmonella* isolates and to explore the mechanisms of resistance.

We noticed that the *bla*_*TEM*_ gene primarily mediated ampicillin resistance, while the *bla*_*CTX-M*_ gene mainly mediated ceftriaxone and cefepime resistance. The results were similar to those reported [33, 45], indicating that the antimicrobial resistance of *Salmonella* was closely related to the presence of antimicrobial resistance genes. However, the 69 ampicillin-resistant *Salmonella* isolates in this study did not carry the *bla*_*TEM*_ gene. The 16 *Salmonella* isolates resistant to ceftriaxone and 13 *Salmonella* isolates resistant to cefepime did not carry the *bla*_*CTX-M*_ gene, indicating that some of the isolates were not consistent between antimicrobial resistance phenotypes and genotypes. This could be other resistance mechanisms or antimicrobial resistance genes mediating the resistance. Therefore, the mechanism of antimicrobial resistance needs to be studied more comprehensively.

In summary, our study provides the first systematic overview of the serotype distribution, MDR trend, and the prevalence of β-lactamase resistance genes in human *Salmonella* isolates from clinical specimens between 2013 and 2018 in Guizhou province of China. The distribution of *Salmonella* serotypes showed regional characteristics. The MDR of *Salmonella* isolates was at a high level and gradually increased from 2013 to 2017. The ARGs of the isolates showed that the *bla*_*TEM*_ gene was the most prevalent, followed by the *bla*_*CTX-M*_ and *bla*_*OXA-1*_ genes. It is important to strengthen this platform for ongoing and enhanced surveillance, which is critical in rapidly increasing antimicrobial resistance.

## Supporting information

S1 Table Antimicrobial resistance genes primer and PCR cycling conditions information. (XLSX)

S2 Table AMR patterns of *Salmonella* isolates in Guizhou province from 2013 to 2018. (XLSX)

S3 Table The serotype distribution of human *Salmonella* isolates in nine cities (prefectures) of Guizhou province. (XLSX)
